# Phylogenetic restriction of plant invasion in drought‐stressed environments: Implications for insect‐pollinated plant communities in water‐limited ecosystems

**DOI:** 10.1002/ece3.7776

**Published:** 2021-07-05

**Authors:** Andrew D. F. Simon, Hannah E. Marx, Brian M. Starzomski

**Affiliations:** ^1^ School of Environmental Studies University of Victoria Victoria BC Canada; ^2^ Department of Biology University of New Mexico Albuquerque NM USA; ^3^ Museum of Southwestern Biology Albuquerque NM USA

**Keywords:** phenology, phylogenetic community ecology, plant invasion, pollinator ecology, temporal niche dynamics

## Abstract

**Background:**

Plant–pollinator community diversity has been found to decrease under conditions of drought stress; however, research into the temporal dimensions of this phenomenon remains limited. In this study, we investigated the effect of seasonal drought on the temporal niche dynamics of entomophilous flowering plants in a water‐limited ecosystem. We hypothesized that closely related native and exotic plants would tend to share similar life history and that peak flowering events would therefore coincide with phylogenetic clustering in plant communities based on expected phenological responses of plant functional types to limitations in soil moisture availability.

**Location:**

Galiano Island, British Columbia, Canada.

**Methods:**

Combining methods from pollinator research and phylogenetic community ecology, we tested the influence of environmental filtering over plant community phenology across gradients of landscape disturbance and soil moisture. Floral resource availability and community structure were quantified by counts of flowering shoots. We constructed a robust phylogeny to analyze spatial and temporal variation in phylogenetic patterns across the landscape, testing the significance of the observed patterns against a randomly generated community phylogeny. Phylogenetic metrics were then regressed against factors of disturbance and soil moisture availability.

**Results:**

Critical seasonal fluctuations in floral resources coincided with significant phylogenetic clustering in plant communities, with decreasing plant diversity observed under conditions of increasing drought stress. Exotic plant species in the Asteraceae became increasingly pervasive across the landscape, occupying a late season temporal niche in drought‐stressed environments.

**Main conclusion:**

Results suggest that environmental filtering is the dominant assembly process structuring the temporal niche of plant communities in this water‐limited ecosystem. Based on these results, and trends seen elsewhere, the overall diversity of plant–pollinator communities may be expected to decline with the increasing drought stress predicted under future climate scenarios.

## Graphical abstract

To investigate how environmental factors impact the temporal niche of plant communities in a Mediterranean‐type system, we used a linear mixed effects model to regress phylogenetic metrics against disturbance and soil moisture availability. Plant diversity decreased with drought stress, particularly among exotic species. Based on these results, and trends seen elsewhere, the overall diversity of plant–pollinator communities may be expected to decline with the increasing drought stress predicted under future climate scenarios.

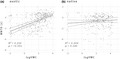

## 1 INTRODUCTION

In Mediterranean‐type ecosystems, late season restrictions in soil moisture availability have a profound impact on plant phenology (Gordo & Sanz, 2010; Spano et al., 2003). In turn, these recurrent episodes of seasonal drought may have a strong influence over pollinator communities (Takkis et al., 2018). Widespread shifts in precipitation patterns are forecasted for these systems under future climate scenarios, including extreme winter rainfall variability and increasingly severe seasonal drought (Seager et al., 2019). Such changes are likely to influence both spatial and temporal dimensions of the pollinator foraging landscape, resulting in shifting abundances and relationships among plant and insect communities.

Increasing drought may cause reductions in floral resource availability, resulting in a lower proportion of flowers containing nectar, fewer flowers per inflorescence, and reduced floral richness in some communities (Phillips et al., 2018; Takkis et al., 2018; Waser & Price, 2016). Drought has also been found to advance flowering phenology among annuals (König et al., 2017), and to cause stress or reduced productivity among perennial shrubs (Pérez‐Camacho et al., 2012). On the other hand, increasing winter precipitation may disproportionately benefit annual herbaceous communities (Pérez‐Camacho et al., 2012). Warming temperatures may also extend the growing season, opening up new temporal niches for exotic species to occupy (Ashbacher & Cleland, 2015; Wolkovich & Cleland, 2014). Many of these findings emphasize the variable response of annual and perennial plant functional types to drought stress, which may have numerous direct and indirect consequences for pollinator ecology.

Diversity among plant functional types can promote niche complementarity in ecological communities (Gross et al., 2007; Gubsch et al., 2011), partitioning resources both spatially and temporally, and thereby increasing overall productivity throughout the year (Schwinning & Kelly, 2013; Wagg et al., 2017; Wolkovich & Cleland, 2011, 2014). In water‐limited ecosystems, annuals tend to germinate during late autumn rains, to concentrate primary productivity during the wettest phase of the year, and to flower in early spring, surviving periods of low soil moisture as seeds. Perennials, by contrast, tend to maintain primary productivity longer, and to flower later, drawing from the tail end of the wet season resource pulse and persisting through drought in vegetative form (Schwinning & Kelly, 2013). Shallow‐rooted annuals develop faster than perennials, gaining priority in acquiring resources (Díaz et al., 2016; Falster & Westoby, 2003; Westoby et al., 2002; Wright et al., 2004), whereas greater root length in perennials may provide a competitive advantage in acquiring resources at deeper depths (Casper & Jackson, 1997; Fargione & Tilman, 2005; Fort et al., 2014). From the point of view of pollinators, the differing life‐history strategies of annuals and perennials may have important consequences, underlying critical seasonal fluctuations in floral resources throughout water‐limited ecosystems. Indeed, functional diversity among plants is known to correlate positively with functional diversity among pollinators, promoting the persistence of diverse plant communities (Fontaine et al., 2005; Papanikolaou et al., 2017). Yet overall species diversity in plant–pollinator communities has been shown to decline under conditions of drought (Hoiss et al., 2015). Understanding the differential response of annuals and perennials to critical resource fluctuations is therefore key to predicting future changes in these communities.

Temporal niches have been postulated as a phenomenon arising from functional trait diversity in plant communities, as fluctuating resources become partitioned by plants with divergent life histories entailing differing regimes of photosynthetic activity and resource uptake to support vegetation and reproduction (Ogle & Reynolds, 2004; Schwinning & Kelly, 2013; Yachi & Loreau, 1999). These dynamics have important implications in the context of plant invasion theory, as changes in the timing and intensity of seasonal drought could open temporal windows of invasion opportunity, benefiting non‐native species adapted to exploit changing environmental conditions at different times of year (Wolkovich & Cleland, 2011, 2014). Researchers have predicted that the late season might present limited opportunity for invasion in water‐limited ecosystems, as non‐native species may not be as well adapted to high levels of drought stress as compared to native species (Alpert et al., 2000; Wolkovich & Cleland, 2014). However, because of the severe restrictions seasonal drought imposes on the vital activities of plants, competitive interactions may be of relatively limited importance to the structure of plant communities in these systems (Kikvidze & Brooker, 2010). Rather than competition, environmental filtering is expected to prevail as the dominant mode of community assembly in these systems, with plant community composition determined primarily by local abiotic constraints. In this context, assuming that drought‐tolerant functional traits are conserved within clades, environmental filtering predicts that a late season temporal niche might indeed become occupied by exotic species insofar as they are phylogenetically related to the native host community.

Environmental filtering has important implications for plant communities in systems affected by seasonal drought, potentially limiting the seasonal availability of floral resources for insect pollinators. To study these dynamics, we investigated the effect of seasonal drought on the phenology of entomophilous flowering plants in the southern Gulf Islands of British Columbia, Canada. We hypothesized that closely related native and exotic plants would tend to share similar life history and that peak flowering events would therefore coincide with phylogenetic clustering in plant communities based on expected phenological responses of plant functional types to limitations in soil moisture availability. To test this hypothesis, we analyzed plant phenology as it varied over the course of the growing season, assessing spatial and temporal overlaps in the local niche breadth of native versus non‐native plants across gradients of disturbance and soil moisture. In dry environments, we predicted that both annuals and perennials would tend to flower early in response to diminishing soil moisture. In wet environments, we predicted both delayed and prolonged flowering among perennials sustained by higher levels of soil moisture. Late flowering was generally expected to correlate with perenniality among related native and exotic plants across all site conditions. We also expected landscape disturbance and drought stress to favor exotic species, increasing late season floral resource availability. Finally, we predicted that the phylogenetic relationships underlying these phenological patterns would reveal effects of environmental filtering, with temporal niches segregated based on similarities in community phylogenetic structure and life history among related native and exotic species.

## 2 METHODS

### 2.1 Study area

Galiano Island lies in the rain shadow of the mountains of Vancouver Island and the Olympic Peninsula, in southern coastal British Columbia, Canada. This region is defined by its cool Mediterranean‐type climate, characterized by mild, wet winters and warm, dry summers (Klassen et al., 2015). Climate normals recorded from nearby Saturna Island (1981–2010) document mean temperatures of 14.5–15.1° and mean monthly precipitation of 37.3–29.6 mm through the driest months of June through September (Environment Canada, 2021). The combined effects of low precipitation, warm temperatures, and a high number of sunshine hours result in an annual moisture deficit during summer months, which varies to some extent depending on precipitation (Moore et al., 2010). These conditions are expected to become more extreme under projected climate change scenarios: increases in winter precipitation and the intensity and duration of seasonal drought are likely in this system (Salathé et al., 2008; Seager et al., 2019; Spies et al., 2010).

### 2.2 Sampling

We analyzed the phenological response of plant communities to seasonal drought using a 2 × 2 factorial study design contrasting four conditions of disturbance and soil moisture availability: (1) dry seminatural environments (woodlands and rock outcrops); (2) wet seminatural environments (wetlands); (3) dry modified environments (disturbed upland areas such as clear cuts); and, (4) wet modified environments (rural areas including gardens, orchards, and fields). Sites were stratified based on available terrestrial ecosystem mapping data (Madrone, 2008), with 6 sites selected per condition (for a total of 24 sites). Limitations in existing site conditions and the logistics of site access resulted in an imbalance in the study design, with 4 sites representing the wet seminatural condition versus 8 sites representing the dry seminatural condition (and 6 sites representing the other two conditions). This imbalance may potentially limit insight into the effects associated with the wet condition in our study. However, our analyses incorporate soil moisture availability as a continuous variable, thereby overcoming some of the limitations associated with the imbalance in categorical analyses. Sites ranged in size from 0.21 to 6.3 ha and varied in their proximity from 650 m to >23 km (Appendix S1) due to limitations in site access and the narrow geography of the island. While grouped site conditions circumscribed similar habitat types based on common soil moisture regimes, disturbance conditions were diverse, including forestry, fire, landscaping, and other anthropogenic effects. Commonalities between site conditions are reflected in the similarity of vegetation communities, as shown in ordination plots (Appendix S2).

To sample floral resource availability (FRA), we randomly distributed 6–8 (2 × 15 m) belt transects throughout each site, using balanced acceptance sampling methods to ensure a random yet spatially balanced distribution of transects (van Dam‐Bates et al., 2018). The number of transects was scaled roughly in proportion to the size of each site, with the aim of capturing variability in floral resource availability across the landscape. Floral resource availability was quantified as counts of flowering shoots, recorded for each plant species at 1‐m intervals, and used to estimate relative abundance, with each interval surveyed comprising a 1 × 2 m area spanning both sides of the transect line. Soil moisture was recorded at 5‐m intervals as volumetric water content (%VWC) using a Field Scout Time‐Domain Reflectometry probe. Sampling was conducted on a monthly basis from April through August 2018, resulting in five samples per site, with each sample period elapsing over 13 days. This study design was intended to achieve a high degree of spatial and temporal resolution in our samples while ensuring that fieldwork was logistically feasible.

### 2.3 Modeling

**FIGURE 1:**
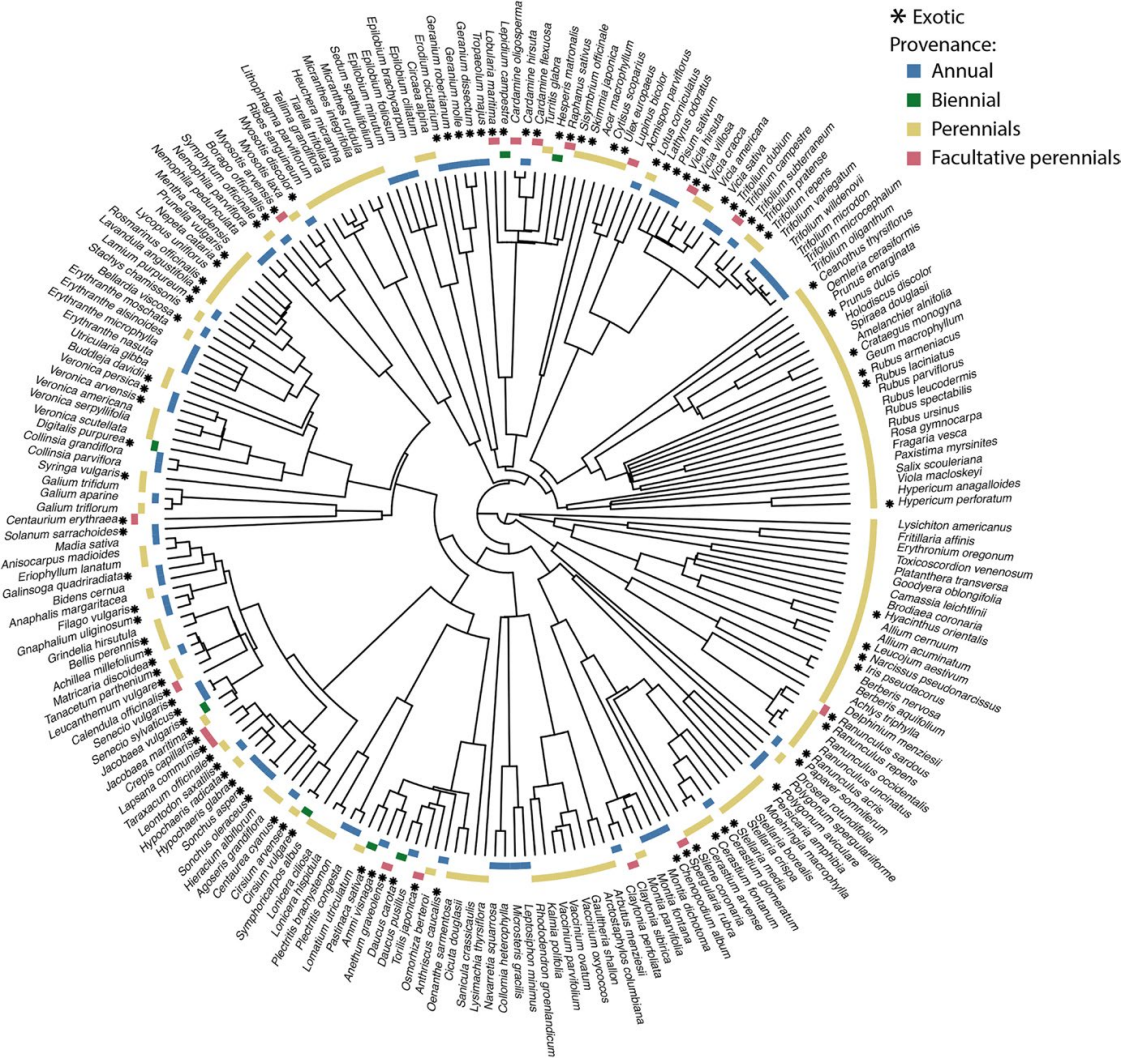
Phylogenetic relationships of entomophilous flowering plants observed on Galiano Island, with labels showing provenance and life history of species

We assembled a phylogeny of seed plants known to Galiano Island based on a previously published robust species‐level phylogeny of 353,185 seed plants derived from GenBank sequence data (Smith & Brown, 2018; Appendix S3). A total of 207 species of entomophilous flowering plants were sampled across sites, 173 of which were represented in the study area phylogeny. This subset of Galiano Island's study area phylogeny comprises the community phylogeny analyzed in this study (Figure 1). Species omitted from this phylogeny due to the lack of sequence data do not demonstrate any taxonomic biases that could affect the results of this study.

We investigated phylogenetic patterns at two spatial scales. First, we pooled transects to assess patterns within each site condition for each sample period. Second, we assessed finer scale temporal patterns across the landscape within each transect for each sample period. To compare phylogenetic patterns between native versus exotic plant assemblages, we compared phylogenetic patterns for each group separately across each community and sample period. Interspecific phylogenetic distance matrices were used to calculate abundance‐weighted mean pair‐wise distance (MPD) and mean nearest taxon distance (MNTD) metrics for community and transect scale matrices. While MPD is a measure of mean phylogenetic distance between all pairs of species in a sample, MNTD measures the average phylogenetic distance from each species to its closest relative. The former metric is sensitive to deep phylogenetic patterns, whereas the latter is more sensitive to terminal branching (Tucker et al., 2017). Both metrics are routinely used in community ecology to test hypotheses about the mean relatedness of taxa within communities.

To determine significance in the phylogenetic structure of community data, we compared observed phylogenetic metrics (MPD and MNTD) calculated for each subset of community data against a null model. The null model was generated by randomly shuffling labels representing taxa across the tips of phylogenies generated based on each subset of community data (randomizations = 999), thereby randomizing assemblages while holding species richness and occurrence frequency constant (Kembel et al., 2010). Standardized effect sizes (*z*) resulting from null model tests were analyzed for each sample period and site condition, to determine the significance of phylogenetic dispersion patterns in community data. Positive *z* values are associated with phylogenetic over‐dispersion; negative values with phylogenetic clustering. The R packages “pez” (Pearse et al., 2015) and “picante” (Kembel et al., 2010) were used to implement community phylogenetic diversity analyses.

Transect‐scale phylogenetic metrics were regressed against environmental variables with linear mixed‐effects models (LMM) using the R package “nlme” (Pinheiro et al., 2021). Random slope‐intercept models were fit by incorporating phylogenetic metrics as a response to log‐transformed soil VWC and categorical factors of disturbance (fixed effects), with transects nested within sites (random effects). Models failed to converge using mean pair‐wise distance (MPD *z*), so mean nearest taxon distance (MNTD *z*) was adopted for these analyses. To test differences in soil moisture availability between sites, random intercept LMMs were fit using the same nested random effects structure, incorporating log‐transformed soil VWC as a response to site conditions as fixed effects (Pinheiro et al., 2019).

Finally, we fit negative binomial generalized linear mixed‐effects models (GLMMs) incorporating counts of flowering shoots as a response to site conditions (fixed effects) and transects nested within sites (random effects) to test differences in floral resource availability (FRA) between site conditions. Similar models were fit for each site condition and sample period to test differences in the contributions of native versus exotic species to FRA and to test differences in FRA contributed by different plant functional types within clades. These models were implemented using the R package “lme4,” and “glmmTMB” to address issues of zero‐inflation (Bates et al., 2015; Brooks et al., 2017). The best models were selected based on AIC test scores (R Core Team, 2019), assuming ΔAIC of 2.0 as a threshold for model improvement (Burnham & Anderson, 2002). Model effects are reported as Incidence Rate Ratios (IRR), and as marginal *R* ^2^ values (variance explained by fixed effects) where appropriate. Marginal effects were calculated using “sjPlot” (Lüdecke, 2018). Statistical analyses were implemented in R Version 3.6.0 (R Core Team, 2019).

## 3 RESULTS

**FIGURE 2:**
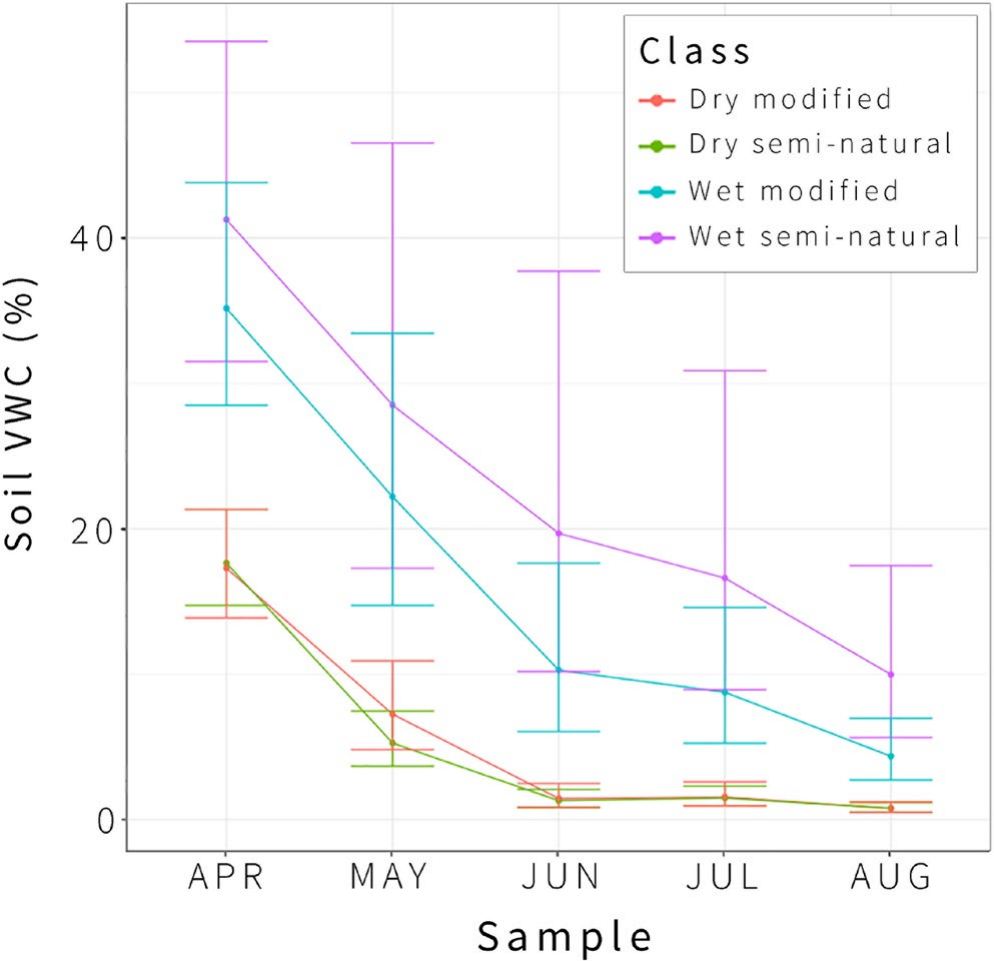
Mean soil moisture across each site class from April through August, shown with 95% confidence intervals (LMM estimates). Soil moisture was estimated as volumetric water content (%VWC), a ratio of water to soil (m^3^/m^3^), using a TDR probe. All differences in wet versus dry site conditions are significant at *p *= <.001–.001

The onset of seasonal drought was exhibited by diminishing soil moisture across all site conditions (Figure 2). Dry seminatural and dry modified sites declined to 1.5 ± 1.5% Volumetric Water Content (VWC) by June, with similarly low soil moisture conditions sustained throughout the rest of the growing season. Wet seminatural sites sustained relatively high soil moisture, declining from 20.6 ± 1.1% VWC in June to 10.6 ± 1.2% VWC in August. Wet modified sites were comparatively dry yet significantly wetter than dry sites from June (10.9 ± 1.5% VWC) until August (4.6 ± 1.5% VWC).

### 3.1 Plant community phenology

**FIGURE 3:**
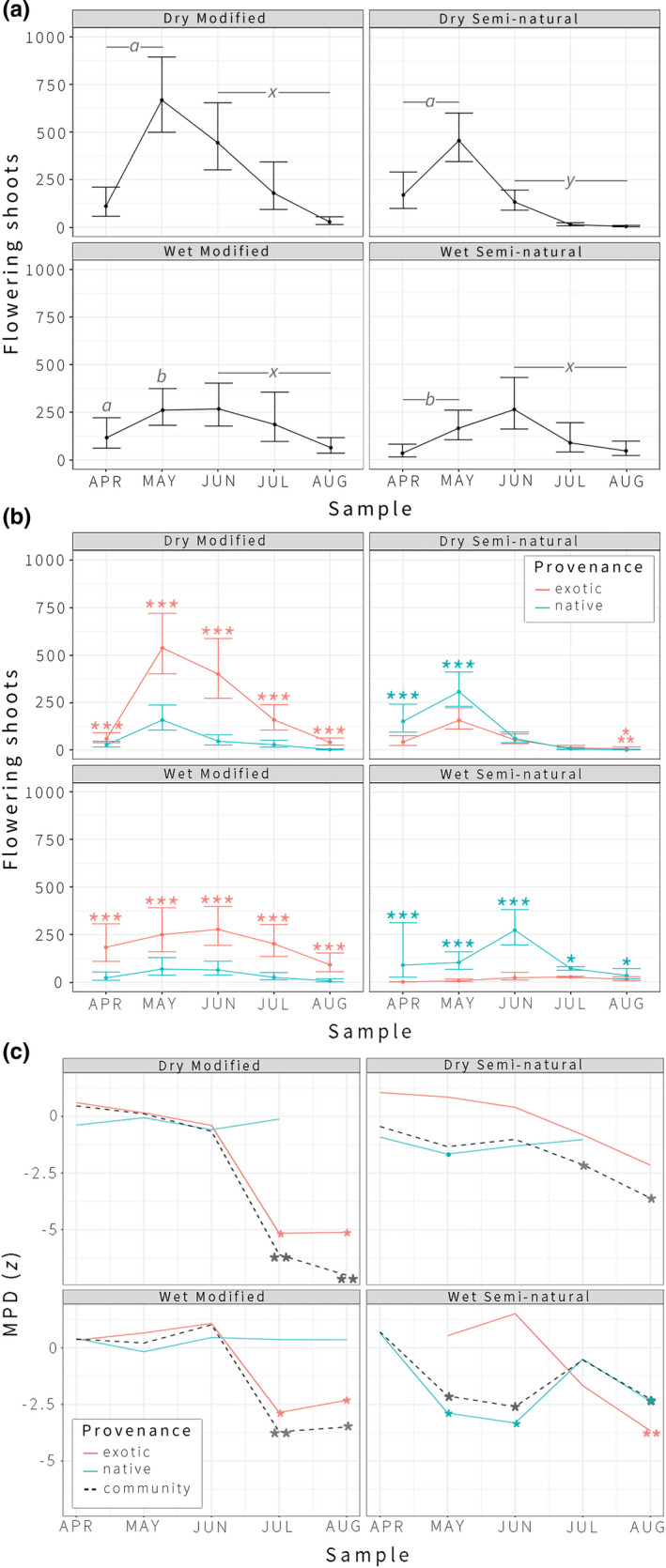
(a) Model outputs of counts of flowering shoots across habitats and samples, with 95% confidence intervals. Significant differences between site conditions, both for independent samples and across sample ranges, are marked with letters (*a* is significantly different from *b*, *x* significantly different than *y*). All differences significant at *p *= <.001–.02. (b) Model outputs of counts of flowering shoots among native versus exotic plants across habitats and samples, with 95% confidence intervals. Asterisks indicate significant differences between native versus exotic FRA at * (*p* < .05) and *** (*p* < .001). ⁂ indicates that significance could not be calculated because only exotic species are present. (c) Standardized effect size of Mean Pairwise Distance (MPD *z*) calculated for native and exotic plant assemblages across site conditions and samples at the community level; negative values indicate increasingly clustered plant assemblages. Significance indicated at * (*p* < .05), and ** (*p* < .01). A bullet (•) marks a near‐significant clustering event (*p =* .056)

Plant community phenology varied significantly across habitat types and sample periods, resulting in distinct peaks and declines in floral resource availability (FRA) over the course of the growing season (Figure 3a). Floral resource availability peaked early in dry seminatural and dry modified habitats, which supported significantly more FRA than wet seminatural habitats from April through May. Floral resource availability peaked later (in June) in wet seminatural and wet modified habitats. Both wet and disturbed conditions generally sustained FRA later into the season, with wet seminatural, wet modified, and dry modified habitats supporting significantly more FRA than dry seminatural habitats from June through August. By August, significantly higher FRA was found in wet modified, wet seminatural, and dry modified habitats versus dry seminatural environments, in rank order of abundance (Figure 3a).

Disturbance was also a critical factor promoting floral resource availability (FRA) among exotic plants (Figure 3b). The highest levels of FRA were found among exotic plants in dry modified environments in May and June, driven largely by the invasive Scotch broom (*Cytisus scoparius*). Declining soil moisture availability was strongly correlated with an increase in FRA among exotic plants in most environments, with significantly higher FRA found among exotic versus native plants overall from June through August. By August, FRA was greatest among exotic plants in most environments, with FRA among native plants diminishing to less than a quarter of the FRA found among exotics across the landscape (IRR_FRA.EXO_. = 0.23_FRA.NAT_., *p* < .001), and FRA sampled exclusively among exotics in dry seminatural habitats. Wet seminatural habitats, by contrast, sustained significantly higher levels of FRA among native plants throughout the growing season (IRR_FRA.NAT_. = 2.53_FRA.EXO_., *p* = .047).

### 3.2 Spatial and temporal niche dynamics

We found patterns of phylogenetic clustering in plant community phenology at multiple scales, from the community to the transect level, which coincided with major flowering events across the landscape. As summarized in Table 1, approximately 90% of the floral resources sampled during this study were found among 12 orders of flowering plants. Major flowering events coincided with significant phylogenetic clustering at community and transect scales, with related plant species found flowering together in the same transects, representing overlaps in the temporal niche breadth of related native and exotic plant species within different site conditions (Table 1).

**TABLE 1:** Mean observed soil Volumetric Water Content (%VWC), with standard errors, and mean floral resource availability (FRA) for 12 dominant plant orders exhibiting significant clustering in transects (GLMM estimates)

Order	Family	N:E	% FRA	*N*	%VWC	Mean FRA				
						April	May	June	July	August
Apiales	Apiaceae	6:6	3	2	15.2 ± 16.1	4	14*	14*	1	<1
Asterales	Asteraceae*	12:26	18	133	8.2 ± 11.7	14*	13	53*	55**	69**
Boraginales	Boraginaceae*	3:4	6	8	17.1 ± 16.1	7*	60*	12*	4	<1
Brassicales	Brassicaceae* Tropaeolaceae*	2:12	4	7	20.1 ± 10.0	45*	7*	2*	2	<1
Caryophyllales	Caryophyllaceae* Droseraceae Montiaceae* Plumbaginaceae Polygonaceae*	12:7	6	12	16.3 ± 14.7	31*	34*	13*	6	<1
Dipsacales	Caprifoliaceae*	5:0	3	1	7.3 ± 9.0	2	5	5*	1	
Ericales	Ericaceae* Polemoniaceae Primulaceae*	14:0	8	10	23.6 ± 25.6	4	12**	19*	8	<1
Fabales	Fabaceae*	9:14	29	38	8.0 ± 8.7	3	84*	56*	5*	<1*
Geraniales	Geraniaceae*	0:5	1	5	8.7 ± 7.5	2	17*	11*	3	<1
Lamiales	Phrymaceae* Plantaginaceae* Lamiaceae* Lentibulariaceae* Orobanchaceae* Scrophulariaceae*	16:18	7	21	18.3 ± 17.3	18*	41*	23*	12*	2**
Myrtales	Onagraceae*	7:0	<1	2	15.5 ± 17.8		4*	3	2	<1
Rosales	Crassulaceae Grossulariaceae Rhamnaceae Rosaceae*	15:10	8	5	17.4 ± 16.9	6	37*	28*	5*	1

Orders are summarized by family, species richness (native:exotic), their overall (%) contributions to FRA across the landscape, and the number of transects (*n*) within which taxa are clustered. The timing of significant clustering events in transects, and families implicated, are marked with an asterisk *. Two asterisks ** indicate significant clustering at the community level. Mean observed soil VWC reflects the temporal and spatial niche breadth of each clade, with higher values associated with early‐flowering phenology and wetter environments, and lower levels associated with late phenology and drier environments. See Appendix S4 for more details on the proportional representation of Plant Functional Types blooming within each plant order, across all site conditions and sample periods.

In May, peak flowering in dry seminatural environments coincided with near‐significant clustering among native plants (MPD *z *= −1.72, *p =* .056) at the community scale. At the transect level, floral resources were concentrated among members of the Boraginaceae, Brassicaceae, Caryophyllaceae, Montiaceae, Phrymaceae, Plantaginaceae, Lamiaceae, and Fabaceae (Table 1). While community‐level clustering in May was near significant only among native species (Figure 3c), significantly clustered transects included related native and exotic plants, the majority of which were herbaceous annuals (Appendix S4). However, there was marked divergence in life history between native and exotic Fabaceae, with the invasive perennial shrubs Scotch broom and gorse (*Ulex europaeus*) blooming among related native and exotic annual herbs—especially in disturbed environments (Appendix S4).

Peak flowering in wet seminatural environments coincided with significant clustering among native plants at the community level in May (MPD *z *= −2.71, *p* = .02) and June (MPD *z* = −2.56, *p* = .04). At the transect scale, floral resources were concentrated among native perennial Ericaceae and Primulaceae (Table 1, Appendix S4). Few exotic species flowered in wet seminatural environments throughout the early and midseason (Figure 3b). By August, however, a diversity of exotic biennial and perennial herbs in the Asteraceae were found within significantly clustered transects in this site condition, as well as many native perennial herbs in the Lamiaceae and Plantaginaceae (Table 1, Appendix S4).

By the late season, the landscape was dominated by the Asteraceae (Table 1). Across all site conditions, significant community‐level clustering coincided with high mean floral resource availability (FRA) in this plant family, including both native and exotic species, with the highest degree of clustering exhibited in dry modified environments in August (MPD *z *= −7.26, *p* <.01). Through July and August, within the Asteraceae, perennial herbs were the most diverse plant functional type flowering most abundantly across the landscape; annuals, biennials, and pauciennials comprised relatively marginal FRA within this family (Appendix S4). In dry seminatural environments, the only plants flowering in August were exotic members of the Asteraceae: the herbaceous biennial bull thistle (*Cirsium* *vulgare*) and herbaceous perennial hairy cat's‐ear (*Hypochaeris radicata*). Appendix S5 provides an overview of the relative abundance of plant functional types across site conditions.

### 3.3 Relationship between phylogenetic patterns and environmental variables

**FIGURE 4:**
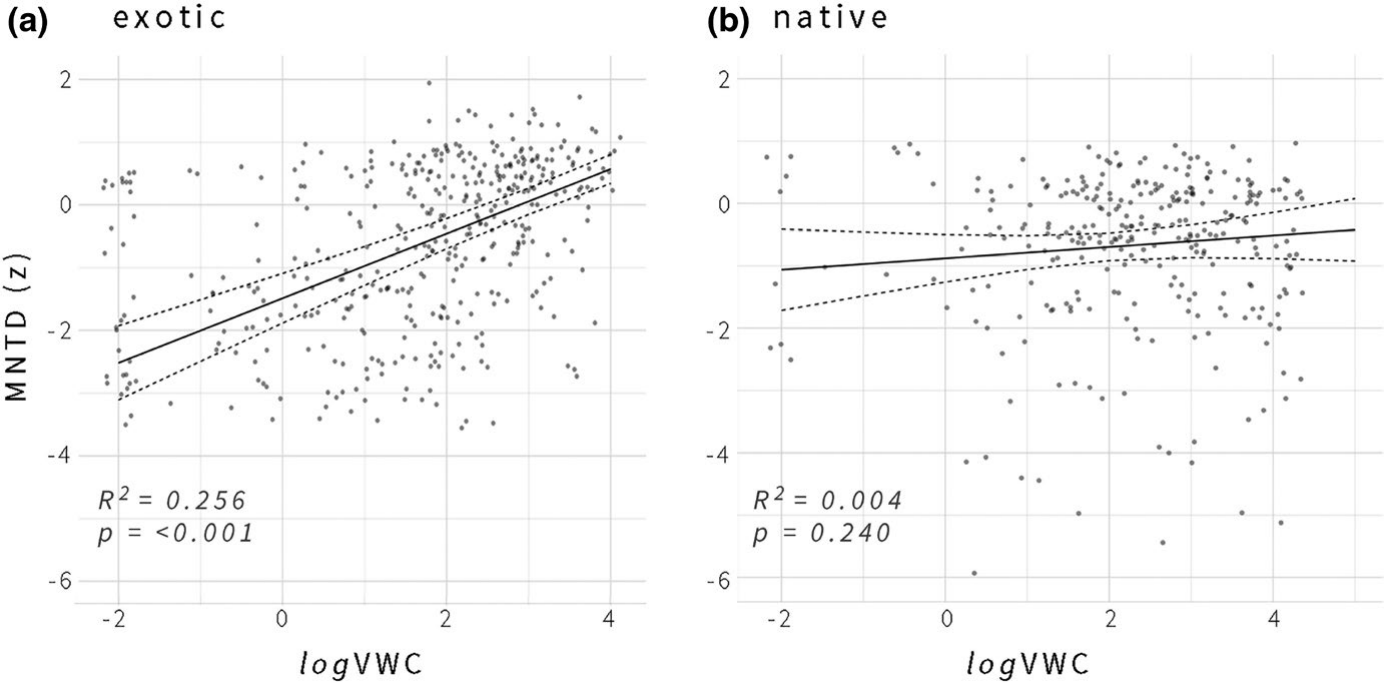
Standardized effect size of the phylogenetic metric mean nearest taxon distance (MNTD *z*), calculated for transects, regressed against log‐transformed soil volumetric water content (logVWC)

Regressing the standardized effect size (*z*) of observed transect‐level Mean Nearest Taxon Distance (MNTD) against *log* Volumetric Water Content (VWC) showed significant phylogenetic clustering among exotic plants as a function of diminishing soil moisture (Figure 4). This relationship is significant when all taxa are pooled, including both native and non‐native species (IRR_logVWC _= 0.30, CI = 0.19–0.42, *p* < .001, *R* ^2^ = .131), yet is strongest when the exotic cohort of flowering plants is isolated (IRR_logVWC _= 0.52, CI = 0.41–0.62, *p* < .001, *R* ^2^ = .256). The Asteraceae are largely responsible for this effect, as a highly diverse family that flowered prolifically throughout the late season (Table 1). However, the relationship between phylogenetic clustering and soil moisture availability was not found when native plants were isolated for modeling (IRR_logVWC_ = 0.09, CI = −0.06–0.24, *p* = .24, marginal *R* ^2^ = .004). Models incorporating site condition as a random effect did not significantly influence the results nor improve the AIC. Results are presented for top models incorporating site and transect as nested random effects. Disturbance was not a significant predictor of phylogenetic clustering in models, though it remained a significant factor predicting the abundance of exotic species on the landscape, with greater floral resource availability among exotics under high (IRR_H.DIST_. = 3.31_L.DIST_., *p* < .001) and moderate levels of disturbance (IRR_M.DIST_. = 1.8_L.DIST_., *p* = .02). Disturbed site conditions also exhibited greater community‐level clustering than undisturbed site conditions in the late season (Figure 3c).

## 4 DISCUSSION

Environmental filtering is a widespread process driving patterns of functional niche occupancy across plant communities (Li et al., 2018). The results of this observational study provide insights into the spatial and temporal dimensions of this process in a water‐limited ecosystem, consistent with a growing body of empirical evidence demonstrating the physiological basis of eco‐hydrological niche segregation (Araya et al., 2011; Schwinning & Kelly, 2013; Silvertown et al., 2015). As reported for other water‐limited ecosystems (e.g., Pérez‐Camacho et al., 2012; Schwinning & Kelly, 2013), we found that the relative drought avoidance of annuals and drought tolerance of perennials was reflected in the phenological response of plant communities to local fluctuations in soil moisture. Annuals tended to bloom early, especially in dry environments. Perennials were less restricted in their phenology, blooming early, but also later and longer into the growing season, especially in wet environments. Life history was to a large extent conserved within dominant plant families, which tended to exhibit similar phenology depending on local site conditions, occupying distinct temporal niches across soil moisture gradients. These dynamics reflect the principles of phylogenetic niche conservatism, as major flowering events coincided with clustering among related plants sharing similar life history and inferred environmental tolerances.

Theory predicts these nested spatial and temporal patterns, as taxa are regionally expected to be filtered into habitats based on the phylogenetic distribution of adaptive traits and then to further segregate into niches based on competing processes of habitat filtering versus competition among similar species at the community scale (Webb et al., 2002). In this water‐limited system, soil moisture regimes are recognized as the primary (spatial) niche axis around which habitat filtration acts, with communities of dry‐adapted plants establishing in moisture‐shedding sites and wet‐adapted plants establishing in moisture‐receiving sites (Klassen et al., 2015; MacKinnon et al., 1992). Yet within communities, a secondary (temporal) niche axis may be recognized in seasonal fluctuations of soil moisture availability. Under this schema, soil moisture availability locally varies through time, placing abiotic constraints on the phenology of plant communities. Depending on the differential response of plant functional types to these changing conditions, plants are theorized to become segregated into temporal niches (e.g., Araya et al., 2011; Schwinning & Kelly, 2013). To the extent that life history is conserved within clades, such temporal niches may be recognized in patterns of phylogenetic clustering within flowering plant communities.

Environmental filtering was the dominant community assembly process reflected in the phylogenetic structure of temporal niches, with significant clustering coinciding with major flowering events at both the community and transect scale. However, there were instances of divergence in plant functional types within families, which in some cases suggests effects of limiting similarity (e.g., Gause, 1932; MacArthur & Levins, 1967; Mayfield & Levine, 2010). For example, the exotic perennial shrubs *Cytisus scoparius* and *Ulex europaeus* bloomed early alongside related annual herbaceous Fabaceae within dry site conditions. Phylogenetic clustering among early‐flowering Fabaceae in these dry conditions may in part be related to the relationship between nitrogen fixation and water use efficiency in this family (Adams et al., 2016). In other cases, divergence in plant functional types coincided with temporal and spatial niche segregation of related families—as, for example, among the Lamiales, which bloomed early in dry seminatural environments, mostly as annual herbaceous species in the Phrymaceae and Plantaginaceae, and later as perennial herbaceous Lamiaceae and Plantaginaceae, predominantly in wet seminatural environments. Similarly, members of the Fabaceae bloomed early, primarily as annuals in dry seminatural environments, and later as perennials (e.g., *Trifolium repens* L.) in wet modified environments. These patterns suggest that though environmental filtering was a dominant process it did not necessarily preclude effects of limiting similarity in these systems. More importantly, however, they illustrate how competing community assembly processes may sometimes coincide, or become confounded, on different spatial and temporal scales.

Previous research in this region by Marx et al. (2016) provided evidence for both environmental filtering and competition as factors important to the success of invading plant species in this island archipelago. On small islands, the authors found that non‐native plants tended to be more phylogenetically and functionally similar to the native host community, suggesting that, at this scale, environmental filtering due to strong abiotic pressures was the dominant community assembly process. Yet as island size increased, landscapes became more heterogeneous, resulting in increasingly complex abiotic and biotic interactions, with related native and non‐native species exhibiting greater disparity in functional traits (Marx et al., 2016). Our study provides local scale insight into this archipelago scale analysis, demonstrating how habitat diversity at the scale of an island can confound effects of environmental filtering and limiting similarity observed at larger spatial scales, which may not be clear unless temporal niche dynamics are considered.

### 4.1 Phylogenetic restriction of plant invasion under conditions of drought stress

We found a strong correlation between soil moisture availability and patterns of phylogenetic clustering, as quantified by counts of flowering shoots among related plant species. Across many families, a high diversity of plants flowered in the early season in this system, with limited phylogenetic clustering occurring among native species at the community scale when soil moisture levels were highest. However, floral resource availability became increasingly concentrated among closely related plant species under conditions of decreasing soil moisture. Whereas native plant families exhibited broad temporal niche breadth, blooming widely across the soil moisture gradient (e.g., Ericaceae, represented by early‐blooming *Arbutus* and *Arctostaphylos* in dry environments, *Gaultheria*, and *Vaccinium* in mesic to moist environments, and late‐blooming *Kalmia, Rhododendron,* and *Vaccinium* in wet environments), there was a strong effect of phylogenetic clustering as a function of decreasing soil moisture availability among exotic species. The late season temporal niche was dominated by both native and exotic members of the Asteraceae, resulting in significant phylogenetic clustering observed under conditions of increasing drought stress.

Dispersal by wind, summer flowering, and long‐flowering are traits known to contribute to invasion success in water‐limited ecosystems (Lloret et al., 2005) and are typical of regionally occurring members of the Asteraceae (Funk et al., 2009). As predicted, native and non‐native members of this family shared the same late season phenology, though both cohorts were highly diverse in life history, represented across the spectrum of plant functional types. These results indicate that traits other than life history are likely associated with drought tolerance in the Asteraceae, such as the high levels of fructan concentrated as primary storage carbohydrates in this clade (Livingston et al., 2009). That said, annual members of the Asteraceae were relatively marginal in occurrence, and most abundant in disturbed environments where certain species (e.g., *Senecio sylvaticus* L.) are known to be constrained as early successional species within a limited time‐space niche (West & Chilcote, 1968). In dry and wet seminatural (undisturbed) environments, where exotic members of the family (e.g., *Cirsium, Hypochaeris*, *Jacobaea*) are recognized as invasive, non‐native Asteraceae were represented predominately by biennials and perennials. These invasive species are known to modify the diversity, structure, and function of natural habitats, contributing to the decline of many native species found in the region (Dennehy et al., 2011).

In this study, the late flowering of exotic biennial and perennial Asteraceae in both dry and wet seminatural environments suggests that, within this clade, perennial life history may be an adaptive trait supporting plant invasion in undisturbed drought‐stressed environments. These findings provide counter‐evidence to the hypothesis that late season drought might reduce the probability of plant invasion (Alpert et al., 2000; Wigginton et al., 2020; Wolkovich & Cleland, 2014). This further suggests that life history may be a predictor of invasion success in the late season, especially insofar as life‐history traits are shared among related native and exotic drought‐tolerant species.

While this observational study only shows correlations between plant community phenology and seasonal fluctuations in soil moisture during one year of fieldwork, the patterns documented in this study are consistent with theory and experimental findings from the literature focusing on water‐limited systems (Araya et al., 2011; Schwinning & Kelly, 2013; Silvertown et al., 2015). Future research should consider a more robust suite of quantitative functional traits in relation to soil moisture gradients, to resolve a better understanding of temporal niche dynamics in this system. Observation of pollinator visitations would also help to clarify the implications of these dynamics for both plant and pollinator communities.

### 4.2 Implications for plant–pollinator communities

Phylogenetic clustering among dominant clades of flowering plants coincided with major flowering events in this system, underlying dramatic seasonal fluctuations in floral resource availability across the foraging landscape. These patterns are consistent with what might be expected given the effects of environmental filtering in water‐limited environments and may have important ecological implications for the future of plant–pollinator communities in these systems.

In the absence of major anthropogenic disturbance events, soil moisture gradients were likely crucial in shaping plant–pollinator interactions across the foraging landscapes of the past, particularly for insects having annual colony cycles such as bumble bees (*Bombus* spp.) which depend on the availability of floral resources throughout the early and late season (Rundlöf et al., 2014; Williams et al., 2012). However, since the mid‐1800s, disturbances associated with European colonization have altered these ecosystems, with land conversion and changing predator–prey dynamics resulting in increased deer browsing pressures, habitat loss, and the introduction of a high diversity of exotic plants (Martin et al., 2011; Marx et al., 2016). Promoted by disturbance, exotic plants now contribute a significant proportion of floral resources in this system. Research has suggested that urban gardens and agricultural systems supporting a diversity of mass‐flowering plants in the late season may be more important than “unimproved” seminatural habitats in sustaining bee communities under conditions of drought (Wray & Elle, 2015). However, the results of this study show that the phylogenetic restriction of plant invasion in drought‐stressed environments has resulted in a concentration of late season floral resources among exotic Asteraceae in this system. The proliferation of this plant family may have had numerous consequences for plant–pollinator networks historically, potentially benefiting generalist pollinator species, or conversely favoring specialists with narrower foraging requirements (Minckley et al., 2013; Thomson, 2016).

Based on the patterns documented in this study, several predictions can be made concerning the temporal niche dynamics of these communities under future climate scenarios. These potential changes highlight the role of phenology in plant invasion, encouraging management actions informed by an understanding of the temporal niche (Wolkovich & Cleland, 2011; Dinis et al., 2020). Given forecasted increases in the intensity and severity of drought, drought‐tolerant families such as the Asteraceae are likely to expand in this system, especially with increasing anthropogenic disturbance. Members of this family, such as *Hypochaeris radicata*, have been observed blooming late in dry seminatural environments, well after summer drought (Appendix S6). This species is capable of vegetative reproduction (Doi et al., 2006), a known correlate of invasion success (Kolar & Lodge, 2001), and may therefore be expected to further benefit from priority effects (Wolkovich & Cleland, 2014). Similar exotic perennials may be expected to increase in abundance under favorable winter moisture regimes, which could result in the competitive exclusion of native annual herbaceous species. Furthermore, in this study, wetlands were host to a low diversity of exotic (entomophilous) plants yet hosted several exotic biennial and perennial Asteraceae in the late season, particularly on the drier margins of these habitats. Given this pattern, microtopographic rises in wetlands may become increasingly prone to invasion with increasing drought stress. This effect, coinciding with the restricting effects of drought stress on native perennial shrub communities (Caldeira et al., 2015; Pérez‐Camacho et al., 2012), could lead to shifts in the quantity and quality of floral resources in these environments. All of the above changes could result in decreasing diversity and overall changes in the composition of plant–pollinator communities, calling on ecologists and land managers to monitor the temporal niche dynamics of drought‐stressed environments.

## CONFLICT OF INTEREST

The authors have no conflicts of interest to claim in the publication of this research article.

## AUTHOR CONTRIBUTIONS

**Andrew D. F. Simon:** Conceptualization (lead); Data curation (lead); Formal analysis (lead); Funding acquisition (supporting); Investigation (lead); Methodology (lead); Project administration (equal); Resources (supporting); Software (lead); Validation (lead); Visualization (lead); Writing‐original draft (lead); Writing‐review & editing (equal). **Hannah E. Marx:** Conceptualization (supporting); Formal analysis (supporting); Methodology (supporting); Software (supporting); Writing‐review & editing (equal). **Brian M. Starzomski:** Conceptualization (supporting); Formal analysis (supporting); Funding acquisition (lead); Methodology (supporting); Project administration (equal); Resources (lead); Supervision (lead); Validation (supporting); Writing‐review & editing (equal).

### OPEN RESEARCH BADGES

This article has earned an Open Data Badge for making publicly available the digitally‐shareable data necessary to reproduce the reported results. The data is available at https://doi.org/10.5061/dryad.905qfttj7.

## Supporting information

Appendix S1‐S6

## Data Availability

Data and R scripts for implementing analyses supporting this study are available from the Dryad Digital Repository: https://doi.org/10.5061/dryad.905qfttj7.
