# Supplementary material for: Phylogenetic restriction of plant invasion in drought‐stressed environments: Implications for insect‐pollinated plant communities in water‐limited ecosystems
Source: Ecol Evol. 2021 Jul 5;11(15):10042–53. doi: 10.1002/ece3.7776 (PMC8328464; doi:10.1002/ece3.7776)
Supplement: Supplementary file 1 — Appendix S1‐S6 [file ECE3-11-10042-s001.docx]

# Appendix S1. Site Parameters

Study sites summarized by condition, area, and the number of transects allocated to each site. The number of transects was scaled roughly in proportion to the area surveyed, in attempt to balance a feasible study design with efforts to capture variation in plant community composition and phenology. Logistical issues resulted in an imbalanced study design, with fewer replicates in wet semi-natural communities and more replicates in dry semi-natural communities.

| **Site** | **Class** | **Area (ha)** | **Transects** |
| --- | --- | --- | --- |
| 150 Shopland | dry modified | 0.46 | 6 |
| Crystal Mountain | dry modified | 0.25 | 6 |
| DL14 | dry modified | 4.06 | 8 |
| DL24 | dry modified | 0.60 | 6 |
| Heritage Forest | dry modified | 6.30 | 8 |
| Stockade Hill | dry modified | 3.42 | 7 |
| Bluffs Park | dry semi-natural | 4.16 | 8 |
| Bodega Ridge | dry semi-natural | 2.12 | 7 |
| MLC woodland seep | dry semi-natural | 0.21 | 6 |
| Mount Sutil | dry semi-natural | 5.43 | 8 |
| Mount Sutil coastal meadow | dry semi-natural | 0.57 | 6 |
| Retreat Island | dry semi-natural | 0.14 | 6 |
| Sticks West coastal meadow | dry semi-natural | 0.98 | 6 |
| Tapovan | dry semi-natural | 1.11 | 6 |
| Cable Bay Farm | wet modified | 0.94 | 6 |
| Catkin Forest Farm | wet modified | 0.82 | 6 |
| Dexter’s place | wet modified | 1.69 | 7 |
| Marben Farm | wet modified | 0.87 | 6 |
| Mount Sutil Valley Farm | wet modified | 0.96 | 6 |
| Reeces’ Field | wet modified | 2.55 | 7 |
| Devina swamp | wet semi-natural | 1.83 | 7 |
| Ecological Reserve | wet semi-natural | 4.21 | 8 |
| Great Beaver Swamp | wet semi-natural | 1.26 | 7 |
| Laughlin Lake | wet semi-natural | 0.34 | 6 |

# Appendix S2. Vegetation Ordination NMDS Plot


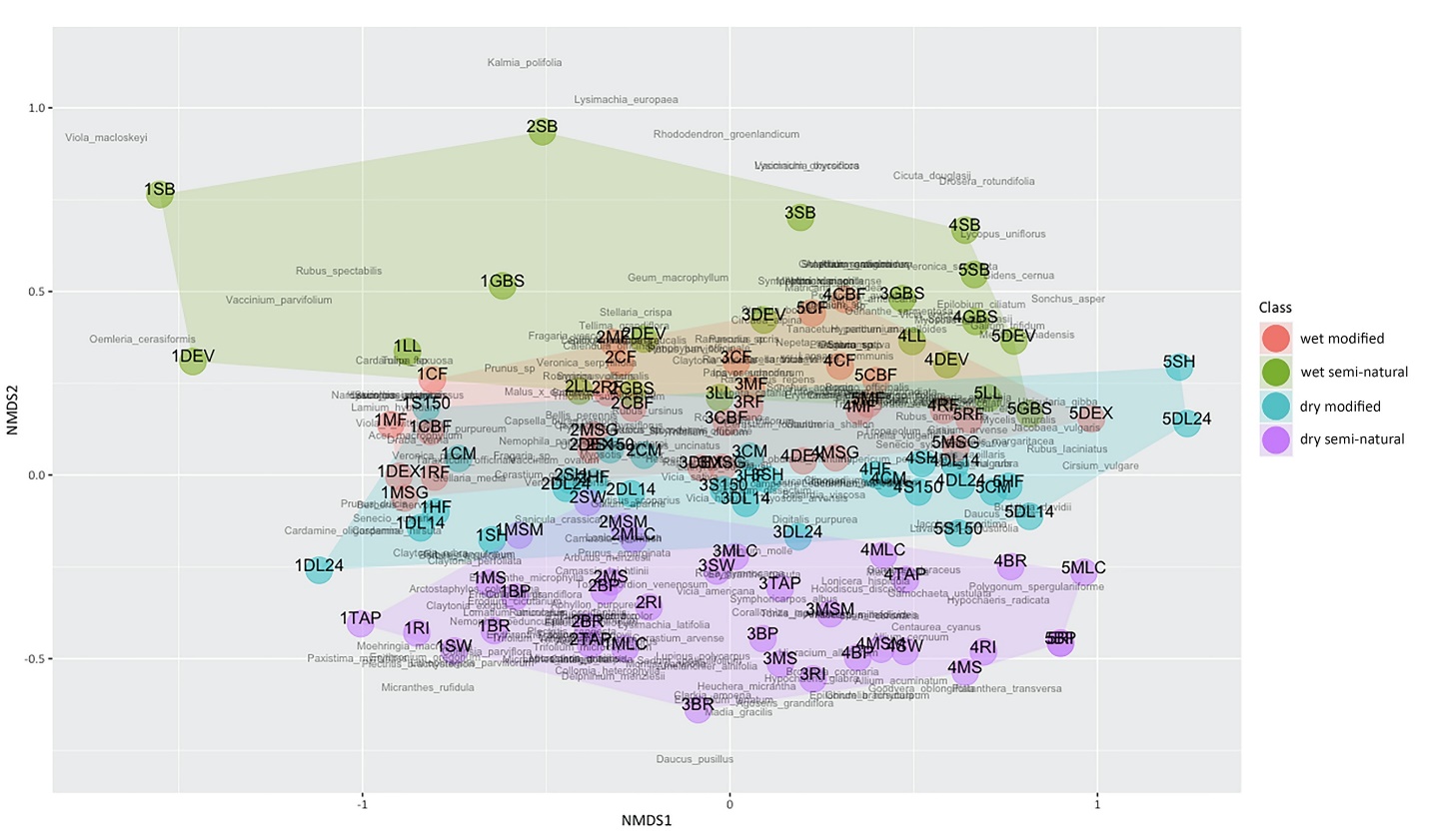


Ordination plot showing similarities between vegetation communities by site (codified by letters) and sample period (numbers).

# Appendix S3. Phylogeny of Galiano Island Seed Plants


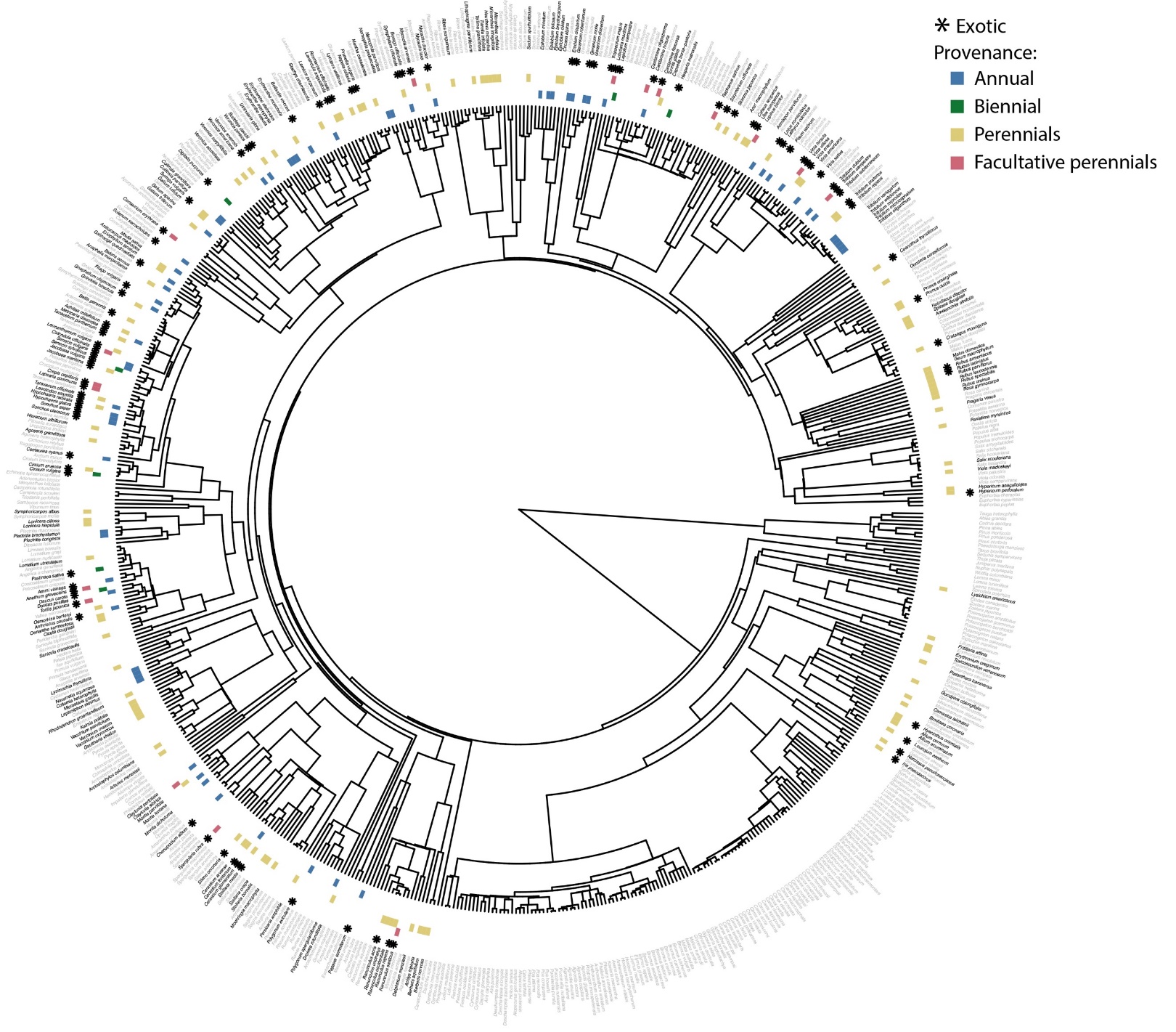


Phylogeny of seed plants known to Galiano Island based on a robust phylogeny of 353,185 seed plants derived from GenBank by Smith & Brown (2018). Of the 717 seed plants recorded in the flora of Galiano Island, 645 species were represented in the reference phylogeny, which were pruned to create this study area phylogeny. Entomophilous flowering plants analyzed in this study are identified in bold, with labels showing provenance and life history of species.

# Appendix S4. Proportional Representation of Plant Functional Types

The following tables summarize proportional floral resource availability among different plant functional types (PFTs) for the dominant plant families sampled over the course of this study. Plant orders are presented alphabetically, grouped by site condition, and species listed by provenance and plant functional type. Significant phylogenetic clustering at the community scale is indicated by three asterisks ⁂; two asterisks ** indicate significant clustering at the transect scale; one asterisk * indicates that plants cooccurred with related species within the same site though they did not cluster within the same transect. The following tables represent >90% of the Floral Resource Availability sampled during this research.

| **Apiales**  (Apiacaee) | | | | | | | | |
| --- | --- | --- | --- | --- | --- | --- | --- | --- |
| Dry semi-natural | | | | | | | | |
| Provenance | PFT | Taxa |  | April | May | June | July | August |
| native | annuals | *Daucus pusillus* |  |  |  | <0.01 |  |  |
| native | perennial herbs | *Lomatium utriculatum*  *Osmorhiza berteroi*  *Sanicula crassicaulis* |  | 1.00 | 1.00 | <0.01** |  |  |
| exotic | pauciennials | *Torilis japonica* |  |  |  | 0.99** |  |  |
| Dry modified | | | | | | | | |
| Provenance | PFT | Taxa |  | April | May | June | July | August |
| native | perennial herbs | *Osmorhiza berteroi* |  |  | 1.00 |  |  |  |
| exotic | biennials | *Daucus carota* |  |  |  |  |  | 1.00 |
| exotic | pauciennials | *Torilis japonica* |  |  |  | 1.00 |  |  |
| Wet semi-natural | | | | | | | | |
| Provenance | PFT | Taxa |  | April | May | June | July | August |
| native | perennial herbs | *Cicuta douglasii*  *Oenanthe sarmentosa*  *Osmorhiza berteroi*  *Sanicula crassicaulis* |  |  | 1.00 | 1.00 | 1.00 |  |
| Wet modified | | | | | | | | |
| Provenance | PFT | Taxa |  | April | May | June | July | August |
| native | perennial herbs | *Osmorhiza berteroi*  *Sanicula crassicaulis* |  | 1.00 | 0.39** | 0.16 |  |  |
| exotic | annuals | *Ammi visnaga*  *Anthriscus caucalis* |  |  | 0.61** | 0.84 |  | 1.00 |
| exotic | biennials | *Daucus carota*  *Pastinaca sativa* |  |  | <0.01 |  | 0.50 |  |
| exotic | pauciennials | *Anethum graveolens*  *Torilis japonica* |  |  |  |  | 0.50 |  |

| **Asterales**  (Asteracae) | | | | | | | | |
| --- | --- | --- | --- | --- | --- | --- | --- | --- |
| Dry semi-natural | | | | | | | | |
| Provenance | PFT | Taxa |  | April | May | June | July | August |
| native | annuals | *Madia gracilis*  *Madia sativa* |  |  |  | 0.16** | 0.16⁂ |  |
| native | perennial herbs | *Achillea borealis*  *Agoseris grandiflora*  *Anisocarpus madioides*  *Eriophyllum lanatum*  *Gamochaeta ustulata*  *Grindelia hirsutula*  *Hieracium albiflorum* |  |  | 0.18 | 0.35** | 0.07⁂ |  |
| exotic | annuals | *Centaurea cyanus*  *Hypochaeris glabra*  *Senecio sylvaticus* |  |  | 0.66 | 0.03** | 0.05⁂ |  |
| exotic | biennials | *Cirsium vulgare* |  |  |  |  | 0.03⁂ | 0.46⁂ |
| exotic | perennial herbs | *Hypochaeris radicata*  *Leucanthemum vulgare*  *Mycelis muralis*  *Taraxacum* sp. |  | 1.00 | 0.16 | 0.45** | 0.65⁂ | 0.54⁂ |
| exotic | pauciennials | *Crepis capillaris*  *Sonchus oleraceus* |  |  |  | 0.01** | 0.04⁂ |  |
| Dry modified | | | | | | | | |
| Provenance | PFT | Taxa |  | April | May | June | July | August |
| native | perennial herbs | *Anaphalis margaritacea*  *Gamochaeta ustulata* |  |  |  |  | <0.01⁂ | 0.01⁂ |
| exotic | annuals | *Filago vulgaris*  *Leontodon saxatilis*  *Senecio sylvaticus*  *Senecio vulgaris*  *Sonchus asper* |  | 0.04 |  | 0.01** | 0.04⁂ | <0.01⁂ |
| exotic | biennials | *Cirsium vulgare*  *Jacobaea vulgaris* |  |  |  |  | 0.05⁂ | 0.19⁂ |
| exotic | perennial herbs | *Bellis perennis*  *Cirsium arvense*  *Hypochaeris radicata*  *Jacobaea maritima*  *Leucanthemum x superbum*  *Leucanthemum vulgare*  *Mycelis muralis*  *Sonchus arvensis*  *Taraxacum* sp. |  | 0.96** | 1.00 | 0.98** | 0.66⁂ | 0.56⁂ |
| exotic | pauciennials | *Crepis capillaris* |  |  |  | <0.01** | 0.25⁂ | 0.23⁂ |
| Wet semi-natural | | | | | | | | |
| Provenance | PFT | Taxa |  | April | May | June | July | August |
| native | annuals | *Bidens cernua* |  |  |  |  |  | 0.01⁂ |
| native | perennial herbs | *Anaphalis margaritacea* |  |  |  |  | <0.01⁂ | 0.02⁂ |
| exotic | annuals | *Senecio sylvaticus*  *Sonchus asper* |  |  |  | 0.24** | 0.15⁂ | 0.06⁂ |
| exotic | biennials | *Cirsium vulgare*  *Jacobaea vulgaris* |  |  |  |  | 0.03⁂ | 0.30⁂ |
| exotic | perennial herbs | *Cirsium arvense*  *Hypochaeris radicata*  *Mycelis muralis*  *Taraxacum* sp. |  | 1.00 |  | 0.52 | 0.73⁂ | 0.52⁂ |
| exotic | pauciennials | *Crepis capillaris*  *Lapsana communis* |  |  |  | 0.24 | 0.09⁂ | 0.08⁂ |
| Wet modified | | | | | | | | |
| Provenance | PFT | Taxa |  | April | May | June | July | August |
| native | perennial herbs | *Symphyotrichum chilense* |  |  |  | <0.01* |  | 0.10** |
| exotic | annuals | *Centaurea cyanus*  *Galinsoga quadriradiata*  *Gnaphalium uliginosum*  *Leontodon saxatilis*  *Matricaria discoidea*  *Senecio sylvaticus*  *Senecio vulgaris*  *Sonchus asper* |  | <0.01** |  | <0.01** | 0.11⁂ | 0.24⁂ |
| exotic | biennials | *Cirsium vulgare*  *Jacobaea vulgaris* |  |  |  |  | <0.01⁂ | 0.02⁂ |
| exotic | perennial herbs | *Achillea millefolium*  *Bellis perennis*  *Cirsium arvense*  *Hypochaeris radicata*  *Leucanthemum x superbum*  *Leucanthemum vulgare*  *Mycelis muralis*  *Sonchus arvensis*  *Tanacetum parthenium*  *Taraxacum* sp. |  | 0.99** | 0.99 | 0.96** | 0.70⁂ | 0.49⁂ |
| exotic | pauciennials | *Calendula officinalis*  *Crepis capillaris*  *Lapsana communis*  *Sonchus oleraceus* |  | <0.01** | 0.01 | 0.03 | 0.19⁂ | 0.15⁂ |

| **Boraginales**  (Boraginaceae) | | | | | | | | |
| --- | --- | --- | --- | --- | --- | --- | --- | --- |
| Dry semi-natural | | | | | | | | |
| Provenance | PFT | Taxa |  | April | May | June | July | August |
| native | annuals | *Nemophila parviflora*  *Nemophila pedunculata* |  | 1.0** | 0.22** | 0.11 |  |  |
| exotic | annuals | *Myosotis discolor* |  |  | 0.78** | 0.89 |  |  |
| Dry modified | | | | | | | | |
| Provenance | PFT | Taxa |  | April | May | June | July | August |
| native | annuals | *Nemophila parviflora* |  | 1.0 | 0.16** | 0.24** |  |  |
| exotic | annuals | *Myosotis discolor* |  |  | 0.84** | 0.53** |  |  |
| exotic | pauciennials | *Myosotis arvensis* |  |  |  | 0.23** | 1.0 |  |
| Wet semi-natural | | | | | | | | |
| Provenance | PFT | Taxa |  | April | May | June | July | August |
| native | annuals | *Nemophila parviflora* |  | 1.0 | 0.86 | 0.08 |  |  |
| native | perennial herbs | *Myosotis laxa* |  |  |  | 0.92 | 1.0 | 1.0 |
| exotic | annuals | *Myosotis discolor* |  |  | 0.14 |  |  |  |
| Wet modified | | | | | | | | |
| Provenance | PFT | Taxa |  | April | May | June | July | August |
| native | annuals | *Nemophila parviflora* |  | 1.0 | 0.10 | 0.02** | <0.01 |  |
| native | perennial herbs | *Myosotis laxa* |  |  |  | <0.01** | <0.01 | 0.05 |
| exotic | annuals | *Borago officinalis*  *Myosotis discolor* |  |  | 0.89 | 0.97** | 0.97 | 0.95 |
| exotic | perennial herbs | *Symphytum officinale* |  |  | 0.01 | 0.01 | 0.02 |  |

| **Brassicales**  (Brassicaceae, Tropaeolaceae) | | | | | | | | |
| --- | --- | --- | --- | --- | --- | --- | --- | --- |
| Dry semi-natural | | | | | | | | |
| Provenance | PFT | Taxa |  | April | May | June | July | August |
| native | perennial herbs | *Turritis glabra* |  |  | 0.06 |  |  |  |
| native | pauciennials | *Cardamine oligosperma* |  | 0.50** |  |  |  |  |
| exotic | annuals | *Cardamine hirsuta* |  | 0.50** | 0.94 | 1.00 |  |  |
| Dry modified | | | | | | | | |
| Provenance | PFT | Taxa |  | April | May | June | July | August |
| native | pauciennials | *Cardamine oligosperma* |  | <0.01 |  |  |  |  |
| exotic | annuals | *Cardamine hirsuta*  *Draba verna*  *Sisymbrium officinale* |  | 0.99** |  |  |  |  |
| Wet semi-natural | | | | | | | | |
| Provenance | PFT | Taxa |  | April | May | June | July | August |
| native | pauciennials | *Cardamine oligosperma* |  | 1.00 |  |  |  |  |
| Wet modified | | | | | | | | |
| Provenance | PFT | Taxa |  | April | May | June | July | August |
| exotic | annuals | *Capsella bursa-pastoris*  *Cardamine hirsuta*  *Draba verna*  *Eruca sativa*  *Sisymbrium officinale*  *Tropaeolum majus* |  | 0.82** | 0.50** | 0.03 | 0.52* | 0.68 |
| exotic | biennials | *Brassica* sp.  *Hesperis matronalis*  *Lepidium campestre* |  | 0.10** | 0.49** | 0.32** | 0.30 | 0.32 |
| exotic | pauciennials | *Cardamine flexuosa*  *Lobularia maritima*  *Raphanus sativus* |  | 0.08** | 0.01 | 0.65** | 0.18 |  |

| **Caryophyllales**  (Caryophyllaceae, Droseraceae, Montiaceae, Plumbaginaceae, Polygonaceae) | | | | | | | | |
| --- | --- | --- | --- | --- | --- | --- | --- | --- |
| Dry semi-natural | | | | | | | | |
| Provenance | PFT | Taxa |  | April | May | June | July | August |
| native | annuals | *Claytonia exigua*  *Claytonia perfoliata*  *Claytonia rubra*  *Polygonum spergulariiforme* |  | 0.94** | 0.67** |  | 0.03 |  |
| native | perennial herbs | *Cerastium arvense*  *Moehringia macrophylla*  *Montia parvifolia* |  | <0.01** | 0.21** | 0.47** |  |  |
| exotic | annuals | *Cerastium glomeratum* |  | <0.01 | 0.06** |  |  |  |
| exotic | perennial herbs | *Silene coronaria*  *Stellaria media* |  | 0.05** | 0.06** | 0.53** | 0.97 |  |
| Dry modified | | | | | | | | |
| Provenance | PFT | Taxa |  | April | May | June | July | August |
| native | annuals | *Claytonia perfoliata*  *Claytonia rubra* |  | 0.58 | 0.85** | 0.01 |  |  |
| exotic | annuals | *Cerastium glomeratum* |  | 0.12 | 0.10** | 0.11** |  |  |
| exotic | perennial herbs | *Cerastium fontanum*  *Silene coronaria*  *Stellaria media* |  | 0.30 | 0.05** | 0.88** | 0.98 |  |
| exotic | pauciennials | *Spergularia rubra* |  |  |  |  | 0.02 |  |
| Wet semi-natural | | | | | | | | |
| Provenance | PFT | Taxa |  | April | May | June | July | August |
| native | perennial herbs | *Drosera rotundifolia*  *Stellaria borealis*  *Stellaria crispa* |  |  | 0.97 | 0.96 | 0.88 | 0.75 |
| native | pauciennials | *Claytonia sibirica* |  |  | 0.03 | 0.01 | 0.12 |  |
| exotic | perennial herbs | *Cerastium fontanum* |  |  |  | 0.03 |  | 0.25 |
| Wet modified | | | | | | | | |
| Provenance | PFT | Taxa |  | April | May | June | July | August |
| native | annuals | *Claytonia perfoliata* |  | 0.01 | 0.02 |  |  |  |
| native | perennial herbs | *Persicaria amphibia* |  |  |  |  | 0.01 |  |
| exotic | annuals | *Cerastium glomeratum*  *Polygonum aviculare* |  | 0.02 | 0.88** | 0.19** | 0.69 | 0.05 |
| exotic | perennial herbs | *Cerastium fontanum*  *Limonium* sp.  *Silene coronaria*  *Stellaria media* |  | 0.97 | 0.10** | 0.81** | 0.30 | 0.95 |

| **Dipsacales**  (Caprifoliaceae) | | | | | | | | |
| --- | --- | --- | --- | --- | --- | --- | --- | --- |
| Dry semi-natural | | | | | | | | |
| Provenance | PFT | Taxa |  | April | May | June | July | August |
| native | annuals | *Plectritis brachystemon*  *Plectritis congesta* |  | 1.00 | 0.99 | 0.31** | 0.67 |  |
| native | perennial shrubs | *Lonicera ciliosa*  *Lonicera hispidula*  *Symphoricarpos albus* |  |  | 0.01 | 0.69** | 0.33 |  |
| Dry modified | | | | | | | | |
| Provenance | PFT | Taxa |  | April | May | June | July | August |
| native | perennial herbs | *Lonicera hispidula* |  |  |  | 1.00 | 1.00 |  |
| Wet semi-natural | | | | | | | | |
| Provenance | PFT | Taxa |  | April | May | June | July | August |
| native | perennial herbs | *Symphoricarpos albus* |  |  |  | 1.00 |  |  |

| **Ericales**  (Ericaceae, Primulaceae, Polemoniaceae) | | | | | | | | |
| --- | --- | --- | --- | --- | --- | --- | --- | --- |
| Dry semi-natural | | | | | | | | |
| Provenance | PFT | Taxa |  | April | May | June | July | August |
| native | annuals | *Collomia heterophylla*  *Leptosiphon minimus*  *Microsteris gracilis* |  |  | 0.19 | <0.01 |  |  |
| native | perennial herbs | *Lysimachia latifolia* |  |  | <0.01 | <0.01 |  |  |
| native | perennial shrubs | *Arctostaphylos columbiana*  *Gaultheria shallon*  *Vaccinium ovatum* | | 1.00 | 0.63** | 0.99** | 1.00 |  |
| native | perennial trees | *Arbutus menziesii* |  |  | 0.18** |  |  |  |
| Dry modified | | | | | | | | |
| Provenance | PFT | Taxa |  | April | May | June | July | August |
| native | perennial shrubs | *Arctostaphylos columbiana*  *Gaultheria shallon*  *Vaccinium ovatum*  *Vaccinium parvifolium* | | 1.00 | 1.00 | 1.00** | 1.00 |  |
| Wet semi-natural | | | | | | | | |
| Provenance | PFT | Taxa |  | April | May | June | July | August |
| native | perennial herbs | *Lysimachia europaea*  *Lysimachia thyrsiflora* |  |  | <0.01⁂ | <0.01⁂ |  |  |
| native | perennial shrubs | *Gaultheria shallon*  *Kalmia polifolia*  *Rhododendron groenlandicum*  *Vaccinium ovatum*  *Vaccinium oxycoccos*  *Vaccinium parvifolium* | | 1.00 | 0.99⁂ | 0.99⁂ | 1.00 | 1.00 |
| Wet modified | | | | | | | | |
| Provenance | PFT | Taxa |  | April | May | June | July | August |
| native | perennial shrubs | *Gaultheria shallon* |  |  |  | 1.00 | 1.00 |  |

| **Fabales**  (Fabaceae) | | | | | | | | |
| --- | --- | --- | --- | --- | --- | --- | --- | --- |
| Dry semi-natural | | | | | | | | |
| Provenance | PFT | Taxa |  | April | May | June | July | August |
| native | annuals | *Acmispon parviflorus*  *Trifolium microcephalum*  *Trifolium microdon*  *Trifolium oliganthum*  *Trifolium variegatum*  *Trifolium willdenovii* |  | 0.09 | 0.41** | 0.29 |  |  |
| native | perennial herbs | *Vicia americana* |  |  |  | <0.01 |  |  |
| native | pauciennials | *Lupinus bicolor*  *Lupinus polycarpus* |  |  | 0.04** | <0.01 |  |  |
| exotic | annuals | *Trifolium dubium*  *Vicia hirsuta*  *Vicia sativa* |  |  | 0.01** | 0.06 |  |  |
| exotic | perennial shrubs | *Cytisus scoparius* |  | 0.91 | 0.54** | 0.64 |  |  |
| Dry modified | | | | | | | | |
| Provenance | PFT | Taxa |  | April | May | June | July | August |
| exotic | annuals | *Trifolium dubium*  *Trifolium subterraneum*  *Vicia hirsuta*  *Vicia sativa* |  |  | 0.08** | 0.21** | 0.31** |  |
| exotic | perennial herbs | *Trifolium pratense* |  |  |  | <0.01** | 0.08** | 0.75 |
| exotic | perennial shrubs | *Cytisus scoparius*  *Ulex europaeus* |  |  | 0.92** | 0.79** | 0.61** | 0.25 |
| exotic | pauciennials | *Trifolium campestre* |  |  |  | <0.01** |  |  |
| Wet semi-natural | | | | | | | | |
| Provenance | PFT | Taxa |  | April | May | June | July | August |
| exotic | annuals | *Vicia sativa* |  |  | 0.46 | 0.84 | 0.07 |  |
| exotic | perennial herbs | *Vicia cracca* |  |  |  |  | 0.93 |  |
| exotic | perennial shrubs | *Cytisus scoparius* |  |  | 0.54 | 0.16 |  |  |
| Wet modified | | | | | | | | |
| Provenance | PFT | Taxa |  | April | May | June | July | August |
| exotic | annuals | *Lathyrus odoratus*  *Pisum sativum*  *Trifolium dubium*  *Trifolium subterraneum*  *Vicia hirsuta*  *Vicia sativa* |  |  | 0.54 | 0.77** | 0.02** |  |
| exotic | perennial herbs | *Lotus corniculatus*  *Trifolium pretense*  *Trifolium repens* |  |  |  | 0.08** | 0.91** | 0.99** |
| exotic | perennial shrubs | *Cytisus scoparius* |  |  | 0.46 | 0.14* |  |  |
| exotic | pauciennials | *Vicia villosa* |  |  |  | 0.01 | 0.07** | <0.01** |

| **Geraniales**  (Geraniaceae) | | | | | | | | |
| --- | --- | --- | --- | --- | --- | --- | --- | --- |
| Dry semi-natural | | | | | | | | |
| Provenance | PFT | Taxa |  | April | May | June | July | August |
| exotic | annuals | *Erodium cicutarium*  *Geranium dissectum*  *Geranium molle* |  | 1.00 | 1.00** | 1.00 | 1.00 |  |
| Dry modified | | | | | | | | |
| Provenance | PFT | Taxa |  | April | May | June | July | August |
| exotic | annuals | *Geranium dissectum*  *Geranium molle*  *Geranium robertianum* |  | 1.00 | 1.00 | 1.00** | 1.00 | 1.00 |
| Wet modified | | | | | | | | |
| Provenance | PFT | Taxa |  | April | May | June | July | August |
| exotic | annuals | *Geranium dissectum*  *Geranium molle* |  |  | 0.95 | 1.00* | 1.00 |  |
| exotic | perennial shrubs | *Rosmarinus officinalis* |  |  | 0.05 |  |  |  |

| **Lamiales**  (Phrymaceae, Plantaginaceae, Lamiaceae, Lentibulariaceae, Orobanchaceae, Scrophulariaceae) | | | | | | | | |
| --- | --- | --- | --- | --- | --- | --- | --- | --- |
| Dry semi-natural | | | | | | | | |
| Provenance | PFT | Taxa |  | April | May | June | July | August |
| native | annuals | *Aphyllon purpureum*  *Collinsia grandiflora*  *Collinsia parviflora*  *Erythranthe alsinoides*  *Erythranthe microphylla*  *Erythranthe nasuta* |  | 0.98** | 0.70** | 0.86 | 0.58 |  |
| native | perennial herbs | *Castilleja hispida*  *Clinopodium douglasii* |  | <0.01 | 0.01 | 0.11 |  |  |
| exotic | annuals | *Lamium purpureum*  *Veronica arvensis* |  | 0.01** | 0.29** | 0.01 |  |  |
| exotic | biennials | *Digitalis purpurea* |  |  |  | 0.02 | 0.01 |  |
| exotic | perennial herbs | *Prunella vulgaris* |  |  |  |  | 0.41 |  |
| Dry modified | | | | | | | | |
| Provenance | PFT | Taxa |  | April | May | June | July | August |
| native | annuals | *Erythranthe alsinoides* |  | 0.04 |  |  |  |  |
| native | perennial herbs | *Clinopodium douglasii* |  |  |  | 0.45* | 0.38** |  |
| exotic | annuals | *Bellardia viscosa*  *Lamium purpureum*  *Veronica arvensis*  *Veronica persica* |  | 0.96** | 1.00 | 0.06* | <0.01** |  |
| exotic | biennials | *Digitalis purpurea* |  |  |  | 0.48* | 0.09** |  |
| exotic | perennial herbs | *Prunella vulgaris* |  |  |  | 0.01 | 0.27** | 0.49 |
| exotic | perennial shrubs | *Buddleja davidii*  *Lavandula angustifolia* |  |  |  |  | 0.26** | 0.51 |
| Wet semi-natural | | | | | | | | |
| Provenance | PFT | Taxa |  | April | May | June | July | August |
| native | perennial herbs | *Lycopus uniflorus*  *Mentha canadensis*  *Stachys chamissonis*  *Utricularia gibba*  *Veronica americana*  *Veronica scutellata* |  |  |  | 1.00 | 0.95 | 0.99⁂ |
| exotic | annuals | *Lamium purpureum*  *Veronica arvensis* |  |  | 1.00 |  |  |  |
| exotic | perennial herbs | *Prunella vulgaris* |  |  |  |  | 0.05 | 0.01⁂ |
| Wet modified | | | | | | | | |
| Provenance | PFT | Taxa |  | April | May | June | July | August |
| native | perennial herbs | *Clinopodium douglasii*  *Erythranthe moschata*  *Stachys chamissonis*  *Veronica americana*  *Veronica scutellata*  *Veronica serpyllifolia* |  | 0.04** | 0.26 | 0.31** | 0.16** | 0.03 |
| exotic | annuals | *Lamium hybridum*  *Lamium purpureum*  *Veronica arvensis* |  | 0.96** | 0.59** | 0.08 | 0.04 |  |
| exotic | biennials | *Digitalis purpurea* |  |  |  | 0.26 | 0.02* |  |
| exotic | perennial herbs | *Agastache* sp.  *Mentha* sp.  *Mentha* *x piperita*  *Monarda* sp.  *Nepata cataria*  *Origanum* sp.  *Prunella vulgaris*  *Salvia* sp. |  |  |  | 0.35 | 0.78** | 0.97** |
| exotic | perennial shrubs | *Syringa vulgaris* |  |  | 0.15 |  |  |  |

| **Liliales**  (Liliaceae, Melanthiaceae) | | | | | | | | |
| --- | --- | --- | --- | --- | --- | --- | --- | --- |
| Dry semi-natural | | | | | | | | |
| Provenance | PFT | Taxa |  | April | May | June | July | August |
| native | perennial herbs | *Erythronium oregonum*  *Fritillaria affinis*  *Toxicoscordion venenosum* |  | 1.00** | 1.00 | 1.00 |  |  |
| Wet modified | | | | | | | | |
| Provenance | PFT | Taxa |  | April | May | June | July | August |
| exotic | perennial herbs | *Tulipa* sp.  *Lilium* sp. |  | 1.00 |  |  | 1.00 |  |

| **Myrtales**  (Onagraceae) | | | | | | | | |
| --- | --- | --- | --- | --- | --- | --- | --- | --- |
| Dry semi-natural | | | | | | | | |
| Provenance | PFT | Taxa |  | April | May | June | July | August |
| native | annuals | *Clarkia amoena*  *Epilobium brachycarpum*  *Epilobium foliosum*  *Epilobium minutum* |  |  | 1.00** | 1.00 | 1.00 |  |
| Dry modified | | | | | | | | |
| Provenance | PFT | Taxa |  | April | May | June | July | August |
| native | perennial herbs | *Circaea alpina* |  |  |  | 1.00 |  |  |
| Wet semi-natural | | | | | | | | |
| Provenance | PFT | Taxa |  | April | May | June | July | August |
| native | perennial herbs | *Circaea alpina*  *Epilobium ciliatum* |  |  |  | 1.00 | 1.00 | 1.00 |
| Wet modified | | | | | | | | |
| Provenance | PFT | Taxa |  | April | May | June | July | August |
| native | perennial herbs | *Circaea alpina*  *Chamaenerion angustifolium*  *Epilobium ciliatum* |  |  |  | 1.00 | 1.00 | 1.00 |

| **Rosales**  (Crassulaceae, Grossulariaceae, Rhamnaceae, Rosaceae) | | | | | | | | |
| --- | --- | --- | --- | --- | --- | --- | --- | --- |
| Dry semi-natural | | | | | | | | |
| Provenance | PFT | Taxa |  | April | May | June | July | August |
| native | perennial herbs | *Fragaria vesca*  *Sedum spathulifolium* |  |  | 0.85 | 0.34 |  |  |
| native | perennial shrubs | *Amelanchier alnifolia*  *Holodiscus discolor*  *Prunus emarginata*  *Rosa gymnocarpa*  *Rubus ursinus* |  |  | 0.15 | 0.65 |  |  |
| exotic | perennial shrubs | *Rubus armeniacus* |  |  |  | 0.01 | 1.00 |  |
| Dry modified | | | | | | | | |
| Provenance | PFT | Taxa |  | April | May | June | July | August |
| native | perennial herbs | *Fragaria vesca*  *Geum macrophyllum* |  |  | 0.03 | 0.01* | <0.01** |  |
| native | perennial shrubs | *Holodiscus discolor*  *Ribes sanguineum*  *Rubus leucodermis*  *Rubus ursinus* |  | 1.00 | 0.97 | 0.90* | 0.13 |  |
| exotic | perennial shrubs | *Rubus armeniacus*  *Rubus laciniatus* |  |  |  | 0.09 | 0.87** | 1.00 |
| Wet semi-natural | | | | | | | | |
| Provenance | PFT | Taxa |  | April | May | June | July | August |
| native | perennial herbs | *Fragaria vesca*  *Geum macrophyllum* |  | 0.03 | 0.02 | 0.03 |  |  |
| native | perennial shrubs | *Oemleria cerasiformis*  *Rubus leucodermis*  *Rubus spectabilis*  *Rubus ursinus*  *Spiraea douglasii* |  | 0.97 | 0.98 | 0.92 | 0.69 | 0.68 |
| exotic | perennial shrubs | *Rubus armeniacus*  *Rubus laciniatus* |  |  |  | 0.05* | 0.31 | 0.32 |
| Wet modified | | | | | | | | |
| Provenance | PFT | Taxa |  | April | May | June | July | August |
| native | perennial herbs | *Fragaria vesca*  *Geum macrophyllum* |  |  | <0.01 | <0.01** | 0.01** |  |
| native | perennial shrubs | *Rosa gymnocarpa*  *Rosa nutkana*  *Rubus parviflorus*  *Rubus spectabilis*  *Rubus ursinus*  *Spiraea douglasii* |  | 0.11 | 0.12** | 0.11 | 0.07 | 0.17 |
| exotic | perennial herbs | *Fragaria* sp.  *Fragaria vesca* |  | <0.01 | 0.04 | <0.01** |  |  |
| exotic | perennial shrubs | *Ceanothus thyrsiflorus*  *Crataegus monogyna*  *Rubus armeniacus*  *Rubus laciniatus*  *Rubus* sp. |  | 0.87 | 0.73** | 0.88** | 0.92** | 0.83 |
| exotic | perennial tree | *Malus x domestica*  *Prunus dulcis*  *Prunus* sp.  *Pyrus* sp. |  | 0.02 | 0.11** |  |  |  |

**Appendix S5. Proportional Floral Resource Availability by Plant Functional Type**

**
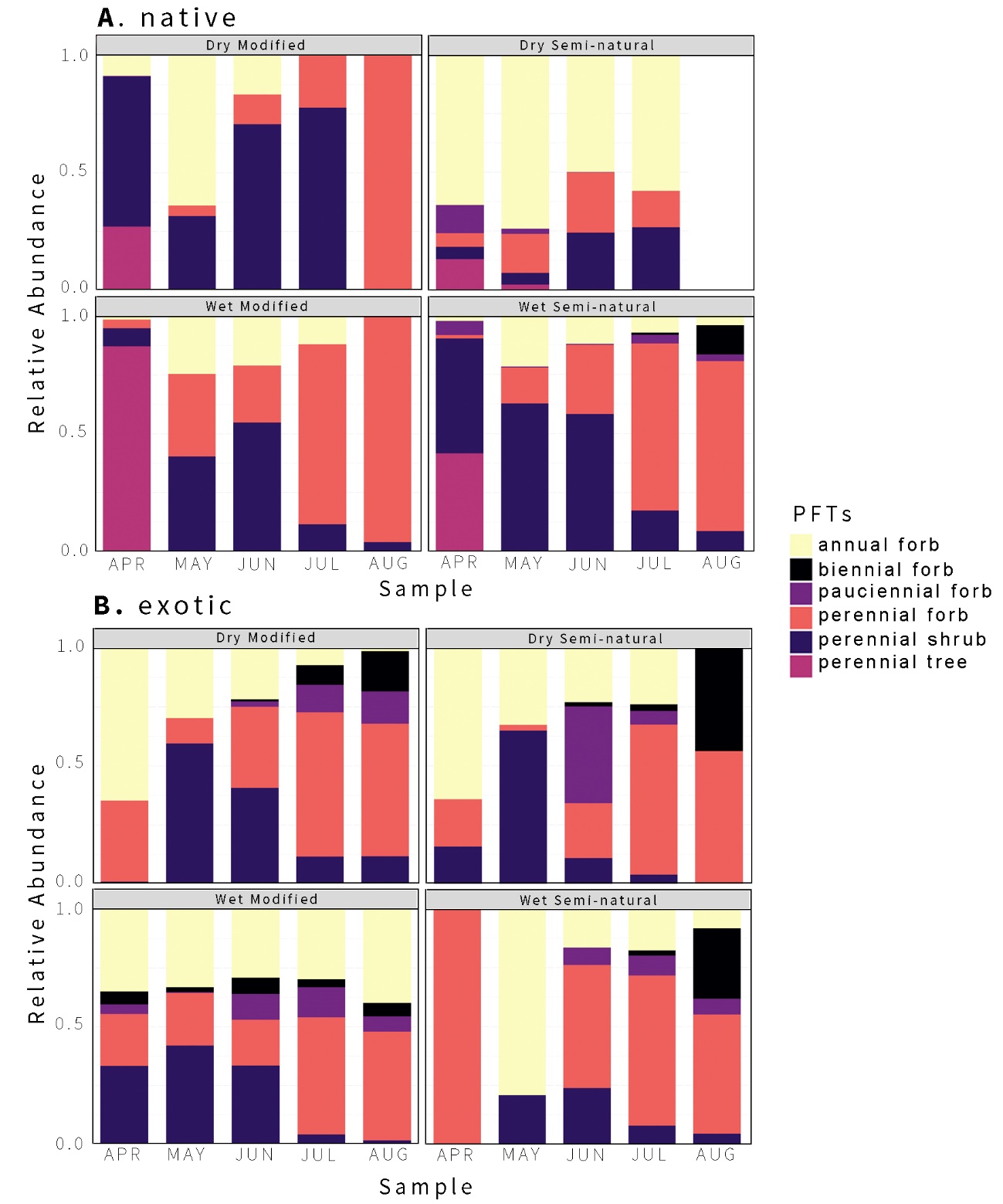
**

Proportional floral resource availability by plant functional type, contrasting (**A**) native and (**B**) exotic plants, for each sample and site condition.

**Appendix S6. *Hypochaeris radicata***


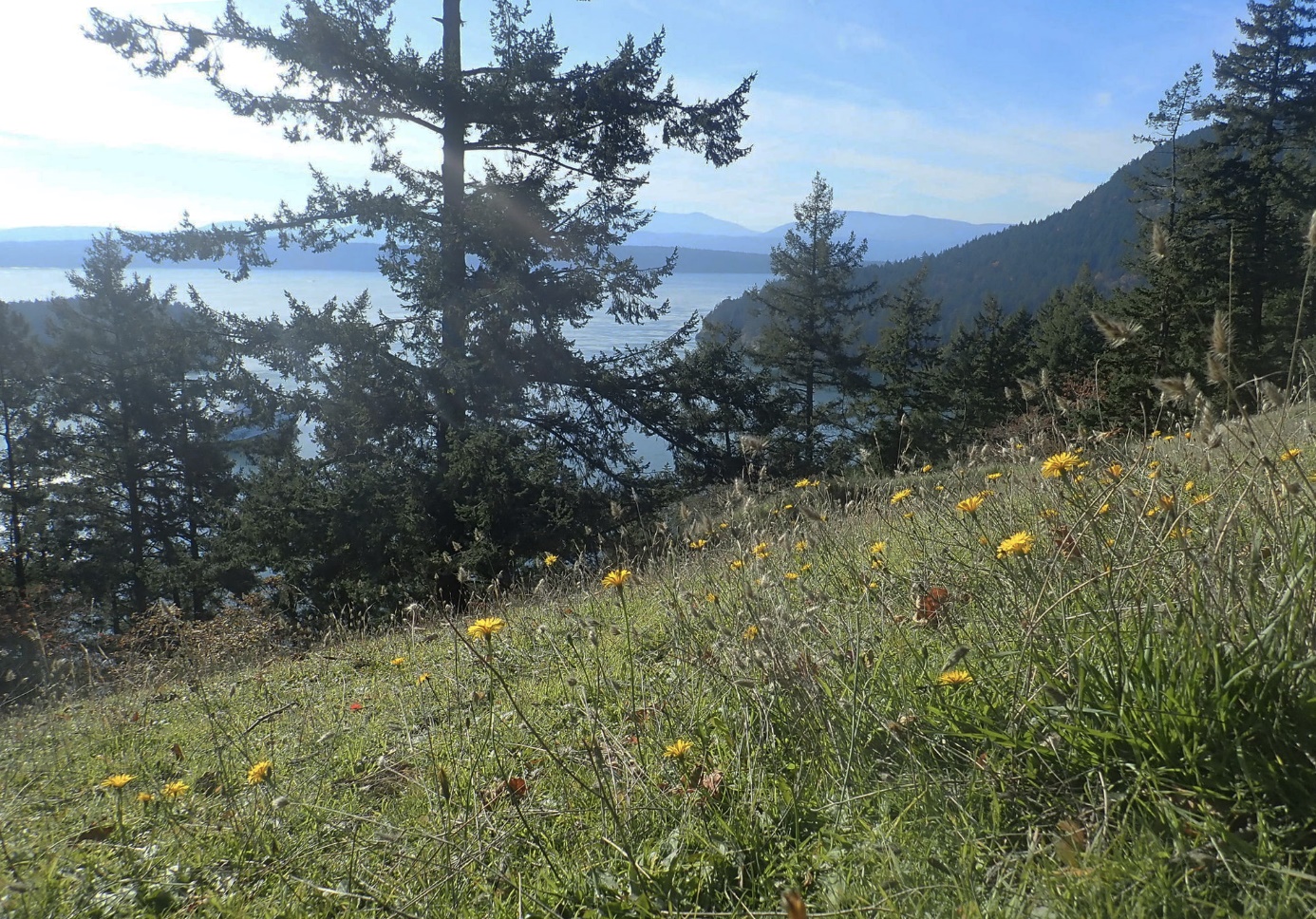


*Hypochaeris radicata* blooming prolifically across slopes host to a diverse community of native annual herbaceous species. November 3, 2019, Galiano Island, BC, Canada.
